# Therapeutic Potential of Hemoglobin Derived from the Marine Worm *Arenicola marina* (M101): A Literature Review of a Breakthrough Innovation

**DOI:** 10.3390/md19070376

**Published:** 2021-06-29

**Authors:** Fareeha Batool, Eric Delpy, Franck Zal, Elisabeth Leize-Zal, Olivier Huck

**Affiliations:** 1 INSERM (French National Institute of Health and Medical Research), UMR 1260, Regenerative Nanomedicine, Fédération de Médecine Translationnelle de Strasbourg (FMTS), 67000 Strasbourg, France; fareeha.batool@etu.unistra.fr; 2 Hemarina SA, Aéropôle Centre, 29600 Morlaix, France; eric.delpy@hemarina.com (E.D.); franck.zal@hemarina.com (F.Z.); elisabeth.leize-zal@hemarina.com (E.L.-Z.); 3 Faculté de Chirurgie-Dentaire, Université de Strasbourg, 8 rue Sainte-Elisabeth, 67000 Strasbourg, France; 4 Pôle de Médecine et Chirurgie Bucco-Dentaire, Hôpitaux Universitaires de Strasbourg, 67000 Strasbourg, France

**Keywords:** *Arenicola marina*, hemoglobin-based oxygen carriers, therapeutic oxygen carriers, M101

## Abstract

Oxygen (O_2_) is indispensable for aerobic respiration and cellular metabolism. In case of injury, reactive oxygen species are produced, causing oxidative stress, which triggers cell damaging chemical mediators leading to ischemic reperfusion injuries (IRI). Sufficient tissue oxygenation is necessary for optimal wound healing. In this context, several hemoglobin-based oxygen carriers have been developed and tested, especially as graft preservatives for transplant procedures. However, most of the commercially available O_2_ carriers increase oxidative stress and show some adverse effects. Interestingly, the hemoglobin derived from the marine lugworm *Arenicola marina* (M101) has been presented as an efficient therapeutic O_2_ carrier with potential anti-inflammatory, anti-bacterial, and antioxidant properties. Furthermore, it has demonstrated promise as a supplement to conventional organ preservatives by reducing IRI. This review summarizes the properties and various applications of M101. M101 is an innovative oxygen carrier with several beneficial therapeutic properties, and further research must be carried out to determine its efficacy in the management of different pathologies.

## 1. Introduction

The existence and maintenance of human life is dependent on oxygen (O_2_) as it is vital for life-sustaining aerobic respiration in humans [1]. At the cellular level, O_2_ acts as the terminal electron acceptor at the end of the electron transport chain within the mitochondrial inner membrane, whereby oxidative phosphorylation results in the synthesis of adenosine triphosphate (ATP), which is necessary for all active metabolic processes [2]. O_2_ is also vital for protein synthesis, maturation of extracellular matrices such as collagen and nitric oxide synthesis, eventually, playing a major role in the regulation of vascular tone and angiogenesis [3]. However, in the presence of O_2_, normal metabolism can also generate reactive oxygen species (ROS). In a wound microenvironment, large amounts of molecular O_2_ are partially reduced to form ROS [3]. ROS are small and highly reactive molecules including superoxide ions and peroxides [4] that influence cell migration, proliferation and angiogenesis [3]. Exacerbation of hypoxic injury after restoration of oxygenation (reoxygenation) is an important mechanism of cellular injury, especially, in transplantation procedures. Cellular hypoxia and reoxygenation are two essential elements of ischemia-reperfusion injury (IRI) [5].

The imbalance between ROS production and the ability of cells and tissue to detoxify these reactive products causes oxidative stress, triggering the production of cell damaging mediators and consequent IRI [6,7,8]. Herein, the excess production or decreased scavenging of ROS has the potential to cause cell injury contributing to human aging and to the pathogenesis of several diseases such as neurodegeneration, diabetes, cancer and atherosclerosis [4]. Oxidative stress is considered to be the major culprit in orchestrating cellular damage in hypoxia-reoxygenation injury and eventual IRI [9,10]

Physiologically, O_2_ is bound reversibly to hemoglobin (Hb), a metalloprotein containing an iron atom at the center of a tetrapyrolic group or porphyrin, contained in red blood cells (RBCs). Hb is a molecule that is responsible for carrying almost all of the O_2_ in the blood, and in humans, it is composed of four subunits (2 alpha and 2 beta globin chains), each of them contains a heme that is able to reversibly bind the O_2_. In the absence of protective enzymes such as superoxide dismutase (SOD) and catalase, Hb is oxidized to methemoglobin (MetHb), thus, compromising its ability to deliver O_2_ [11]. Hb oxidation can compromise efficient O_2_ binding to Hb through the formation of toxic heme degradation products, thereby, hampering optimal tissue perfusion [12]. Sufficient O_2_ supply to tissues is crucial for optimal organ function, the complete failure of which can require an organ transplant. Good tissue oxygenation is mandatory during organ preservation or perioperatively during different major surgical procedures [13,14]. Rigorous research has been carried out over the years in order to find solutions to reach the optimal oxygen requirement during surgery or organ preservation. No single strategy has shown complete success in this context. Over the past decades, artificial O_2_ carriers have been proposed to improve O_2_ supply to tissues [15], including Hb-based O_2_ carriers (HBOCs) and perfluorocarbon-based O_2_ carriers [16,17]. Interestingly, some HBOCs have shown therapeutic promise in maintaining graft/organ vitality and function, and in minimizing the risk of reoxygenation injuries or IRI [18,19]. Also, considering their additional benefits as potential blood substitutes without the inconvenience of crossmatching, their application in maintaining optimum tissue oxygenation is deemed highly appropriate and is under further investigation [20].

Indeed, several mammalian source Hbs have also been tested for their potential as blood substitutes and therapeutic O_2_ carriers [21,22,23,24,25]. Interestingly, Hemopure (HBOC-201) has been approved by the South African drug council for the treatment of anemia. Moreover, this product is allowed to be used in United States in qualified patients to treat severe life-threatening anemia under the FDA’s expanded compassionate use access program [20,26,27,28,29]. Additionally, one randomized, multicenter trial investigation of HBOC-201 in non-cardiac surgery patients indicated that its use reduced RBC transfusions in 43% of patients without notable differences in mortality and serious adverse events, though there was a notable increase in the associated non-serious events (i.e., hypertension and fever) [26]. Unfortunately, while many of these HBOCs showed promise in early clinical trials [15,28,30,31], they eventually failed Phase III clinical trials due to severe adverse effects, such as hypertension, myocardial infarction, stroke, renal damage and tissue toxicity, which are caused by rapid Hb oxidation and scavenging of the vasodilator nitric oxide by Hb [28,32,33,34]. These side effects can be attributed to the removal of intracellular mammalian Hb from the protective environment of the RBC and its release, even the modified forms, into the harsh extracellular environment of the bloodstream [12].

In this context, natural extracellular Hb found in annelids may prove to be advantageous over mammalian Hbs [12,35]. For instance, *Arenicola marina*-derived natural extracellular Hb has been studied in the recent past [35,36,37]. *Arenicola marina’s* Hb (M101, commercially manufactured as HEMO_2_life^®^ by Hemarina (Morlaix, France) has been shown to effectively deliver O_2_ in vivo [38,39] without any signs of oxidation, vasoconstriction, or hypertension [35,40]. Large heme pockets of *Arenicola marina* Hb allow O_2_ to easily escape in passive way [41,42,43]. It is notable that Hb of *Arenicola marina* has a significantly higher O_2_ affinity as reflected by its lower p50 value (7.05 ± 0.93 mmHg) compared to that of human blood, targeting its O_2_ delivery action to a more hypoxic environment [37] (Table 1). However, the p50 of M101 is closely related to the p50 of human HbA inside the red blood cell and its cooperativity is also similar [37].

*Arenicola marina* inhabits the intertidal area in a sulfide-rich environment and is exposed to pronounced fluctuations in environmental conditions [50]. The manufacturing of the product HEMO_2_life^®^ is done under GMP and starts by freezing the worms to create a hemorrhagic shock and to release its extracellular Hb (M101). After successive steps of solid/liquid extraction, purification, filtration and gamma irradiation, the final result is a class III medical device containing M101 [51]. Preclinical studies in rats and hamsters using M101 have shown reduced microvascular vasoconstriction and no significant impact on mean arterial blood pressure compared with other HBOCs that contain bovine or human Hb [40]. Moreover, the biocompatibility and absence of toxicity of HEMO_2_life^®^ has also been evaluated according to international standards (ISO 10993).

In view of the beneficial properties exhibited by M101, the aim of this review is to summarize the outcomes of M101 application to several in vitro, in vivo and human trials and to assess its potential therapeutic and clinical relevance. To perform this review, only studies published in the English language were included and the last search was carried out in August 2020. The following keywords were used for the search: (“M101” OR “HEMO_2_life” OR “Arenicola marina” OR “extracellular hemoglobin” OR “HEMOXYCarrier”) and a systematic literature search was performed in the Pubmed/Medline database. One hundred ninety-one articles were found and after they were reviewed for relevance and repetition, only 16 articles were included in this review.

## 2. Structure of M101

The description of the quaternary structure of M101 and an assessment of its heme content was first carried out in 1996. Multi-angle laser-light scattering electrospray-ionization mass spectrometry [50] gave a molecular mass of 3648 ± 24 kDa and a gyration radius of 11.3 ± 1.7 nm. According to Zal et al., in the reduced condition, the Hb is composed of eight globin chains of molecular masses 15952.5 Da (al), 15974.8 Da (a2), 15920.9 Da (bl), 16020.1 Da (b2), 16036.2 Da (b3), 16664.8 Da (c), 16983.2 Da (dl), 17033.1 Da (d2). In the native Hb, chains b, c, d occur as five disulfide-bonded trimer subunits T with masses of 49560.4 Da (T1), 49613.9 Da (T2), 49658.6 Da (T3), 49706.8 Da (T4), 49724.5 Da (T5). There are two different linker chains, L1 and L2, involved in homo- and heterodimer formation, with a mass of 25174.1 and 26829.7 Da, respectively [45].

**Figure 1:**
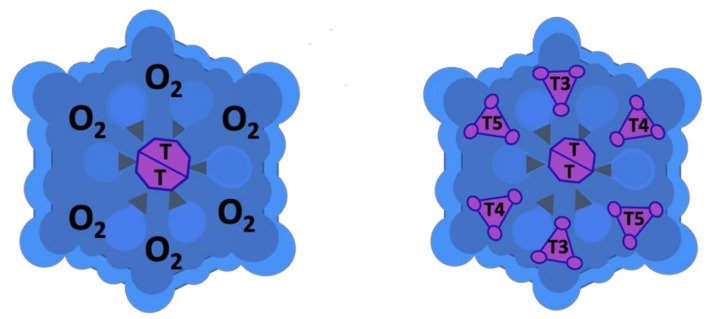
Diagrammatic representation of the structure of *Arenicola marina’s* hemoglobin (M101) molecule.

Thus, the *Arenicola marina* Hb would be composed of 198 polypeptide chains with 156 globin chains and 42 linker chains, each twelfth being in contact with 3.5 linker subunits, providing a total mass of 3682 kDa [50]. The phylogenetic analyses of the *Arenicola marina* globin chains confirmed a more intricate structural organization of this extracellular globin compared to the previous description [50] and have allowed DNA sequencing of *Arenicola marina* globin genes, which assigned the five *Arenicola marina* sequences to the different globin sub-families. The cDNA-derived amino acid sequence exhibits 12 cysteine residues, which is in agreement with previous studies on *Arenicola marina’s* Hb [52]. However, two of the globins were found to be A2 globin chains lacking the cysteine residues proposed to be involved in the binding of hydrogen sulfide by such Hb, which has been linked to the evolution of the Hb sulfide binding function in annelids inhabiting sulfide-rich environments [45,53]. In summary, M101’s quaternary structure comprises two overlapping hexagons, which are approximately 25 nm between parallel sides (face view) and 15 nm thick (profile view) (Figure 1).

## 3. Properties of M101

The *Arenicola marina* Hb M101 can carry up to 156 O_2_ molecules (versus 4 for human hemoglobin) in a saturated state, hence, it exhibits a greater O_2_-binding capacity and antioxidative properties. M101 releases O_2_ according to a simple gradient [35] and without co-factors. M101 can completely inhibit reduction of nitroblue tetrazolium in the presence of superoxide radicals, thereby, demonstrating intrinsic SOD-like activity linked to Cu/Zn metals and an ability to ameliorate oxidative stress [54].

Sulfide concentration in the pore water of the sediment (lugworm burrow) can reach several hundred µmol/L and is considered an important environmental factor for a sediment-dwelling animal such as *Arenicola marina* [55,56]. In the presence of sulfide, vertebrate oxyhemoglobin forms sulfhemoglobin. The inability of *Arenicola marina’s* Hb to form this compound might indicate a biochemical adaptation that is necessary for *Arenicola marina* during exposure to high concentrations of sulfide. The free cysteine on the polypeptide d chains plays a role in this mechanism by inhibiting the formation of sulfhemoglobin and allowing sulfide oxidation. The oxidation product, defined as the brown pigment, and which possesses higher sulfide oxidation activity than hexagonal bilayer Hb, could be due to some modification or breakage of the prosthetic-group-protein linkage during the reaction leading to its formation. However, for *Arenicola marina*, this would be a detoxification mechanism (i.e., sulfide immobilization by the Hb), and not an adaptation to symbiotic life as is the binding of sulfide by vestimentiferan Hb [50]. Interestingly, M101 is able to work in a broad range of temperatures (4 °C to 37 °C) as opposed to other therapeutic oxygen carriers manufactured using vertebrate Hb, which only work at 21 °C to 37 °C [19,57]. In vivo, studies carried out on mice and rats showed that the half-life of M101 molecule in circulation is 2.5 days [38]. The important properties of M101 have been listed below (Table 2).

## 4. Applications of M101

M101 has already demonstrated its efficacy as a supplement to the solution for graft/organ preservation under static and perfusion conditions during organ transplant. Several in vivo preclinical trials involving different vital organs, for instance, heart [62], kidneys [58,63], liver [64], pancreas [65], lungs [66] have exhibited its ability to maintain and improve graft viability and function. Furthermore, its safety and efficiency has also been established in the human trial OXYOP (Clinical Trial Registry No. NCT 02652520) involving a kidney transplant procedure where HEMO_2_life^®^ as the class III medical device containing M101 (1 g/L) was added to a preservation solution [51]. Interestingly, no immunological, allergic or prothrombotic effects were observed with M101 application. Furthermore, addition of M101 decreased the delayed graft function (DGF) and improved the renal function [51]. This confirmed the safety and efficacy of M101 for its potential clinical applications. M101 has also been proposed as an instant blood substitute for wounded US army soldiers [67].

## 5. M101 and Conventional Preservative Techniques

Hypothermic cold storage (CS) is a conventional strategy based on the principle of metabolism reduction with temperature to minimize ischemic injuries during organ preservation [68], however, even slow metabolism requires O_2_ [69,70]. Furthermore, the delayed O_2_ delivery to ischemic tissue during CS could also aggravate oxidative stress if the cells fail to restore oxidative respiration [71]. Therefore, the use of an O_2_ carrier during the entire preservation procedure could be protective, allowing cold-preserved cells to maintain their ATP reserve, thus, establishing a balanced energy metabolism at reperfusion and coping with the sudden influx of O_2_ without causing oxidative stress.

In vitro, M101 supplementation to a range of solutions used in the clinic has demonstrated utility in cold-stored cultured cells. Furthermore, in vivo, M101 addition to University of Wisconsin (UW) and histidine-tryptophane-ketoglutarate (HTK) solution [72] has shown an improvement of graft function and reduction in DGF [57,63].

However, the tissue oxygenation ability of M101 is in accordance with the physiological needs as O_2_ binding and release occurs passively in a simple O_2_ gradient. The intrinsic Cu/Zn-SOD activity of M101 is crucial for the prevention and management of IRI [58,63,73]. Thus, the simple addition of M101 to CS presents excellent potential for clinical translation. Besides static CS, machine preservation (MP) of grafts has attracted mounting interest as it exhibits improvement in graft quality and function [74,75,76]. Its ability to get rid of metabolites and cellular waste produced during ischemia has been proposed as the reason for its beneficial effects. However, the presence of these products at reperfusion is most likely associated with the intense activation of the innate immune pathway [77,78]. In this context, M101 supplementation to MP for uniform and efficient O_2_ delivery to the entire graft could promote organ protection because of the synergistic beneficial effects of M101 and MP [79,80].

## 6. M101 as an Extracellular O_2_ Carrier

Conventional preservative techniques provide O_2_ in excess, which could induce oxidative stress. On the contrary, M101 has the unique property of providing O_2_ against a gradient, according to the physiological needs of the cell, thus, eliminating the risk of hyperoxia and oxidative stress [19,35,38]. M101 supplementation increases cellular ATP production, thus, it efficiently maintains the metabolic processes, and protects mitochondria by decreasing the need to switch from mitochondrial respiration to anaerobic glycolysis. The ability of M101 to maintain high ATP levels during the preservation process may also benefit the restoration of energy homeostasis upon reperfusion due to less metabolic stress on oxidative pathways [57]. M101 remains stable in different organ preservation solutions of varying ionic compositions and osmolarities and demonstrates its O_2_ carrying and antioxidant (SOD) properties [35]. So far, M101 application in vitro, in vivo and clinically has shown great promise (Table 3 and Table 4).

**Figure 2:**
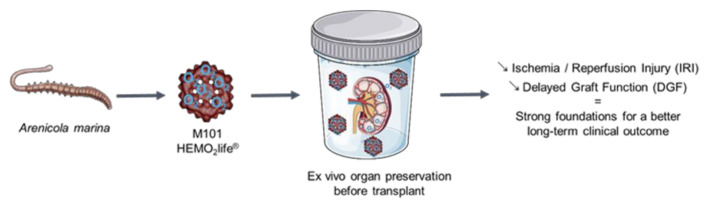
The use of Hb derived from *Arenicola marina* (M101) as a graft preservative can reduce ischemic reperfusion injury and delayed graft function, thus, improving graft vitality and a successful transplant outcome.

Hence, M101 improves graft vitality by promoting tissue oxygenation, maintaining tissue integrity, reducing IRI, inflammation and fibrosis (Figure 2).

## 7. Perspectives

Owing to its interesting O_2_ carrying capacity, the potential of M101 as an O_2_ transporter for numerous pathologies involving hypoxia is worth exploring. Furthermore, its prospects as a therapeutic O_2_ carrier for conditions involving massive hemorrhage, for instance, accidents, terrorist attacks and war injuries should be investigated. M101’s potential application as a temporary or intermittent use alternative for reducing the adverse side-effects associated to pathologies requiring frequent blood transfusions should be further investigated. Additionally, with the recent evolution of its anti-inflammatory and anti-bacterial properties, further research is essential to examine its therapeutic potential for the treatment of inflammatory and infectious diseases. Besides, its incorporation into scaffolds should be tested to establish its feasibility in clinical applications.

Interestingly, the product HEMO_2_life^®^ has been used to preserve a face transplant during a full-face re-transplantation. The patient was transplanted in January 2018 with a graft preserved with HEMO_2_life^®^. The surgery was a success and 30 months post-surgery, the patient is safe and the graft has not been rejected [83]. Recently, M101 has also been proposed as a “molecular respirator”, a potential tool to help COVID-19 patients in their struggle with hypoxemia [84]. Testing this hypothesis further could be instrumental in developing a potential therapeutic strategy to combat the COVID-19 crisis.

## 8. Conclusions

Therapeutic O_2_ carriers could be used as graft/organ preservatives and could also present an alternative in cases requiring frequent blood transfusion. However, extensive investigation is warranted to establish their safety and efficacy, especially for their potential clinical application. Moreover, M101, being an efficient O_2_ transporter, may be a promising medical product with therapeutic potential for the management of several pathologies.

## Figures and Tables

**Table 1:** Properties of *Arenicola marina’s* Hb.

Properties	Hb of *Arenicola marina*	References
Molecular weight	3600 kDa	[35,44,45]
Size	15 by 25 nm	[44,46]
Shape of central piece	Ellipsoid (hexagonal bilayer)	[46]
O_2_ binding sites	156	[35,44]
Heme pockets	large	[36,47]
Auto-oxidation rate k_ox_ (h^−1^)	0.014	[35,48]
Redox potential E_o_ (mV)	−50	[48]
p50	7.05 ± 0.93 mmHg	[40,49]
n50	2.54 ± 0.23	[29]

**Table 2:** Comparison between the properties of *Arenicola marina’s* Hb and human Hb under human physiological conditions (pH 7–8).

Properties	*Arenicola marina’s* Hb	Human Hb	References
Colloid oncotic pressure	1 mm Hg		[35,40]
Size/weight	3600 kDa	64458 Da	[35]
Half life	50 h	few seconds	[35,40]
Working temperature range	Broad (4 °C to 37 °C)	37 °C	[35,40]
p50 (mmHg)	7.05 ± 0.93	8–18	[35,40,58]
n50	2.54 ± 0.23	2.3–3.0	[35,40,59]
Bohr coefficient	−0.5	−0.48	[35,40,60]
Δ H (KJmol^−1^)	−19	−50.7	[35,40,61]
COP (mmHg)	1.0	nd	[40]
Viscosity (cP)	1.23	nd	[40]
SOD activity (U/mg Hb)	3.53 ± 0.02	0	[40]
Cyanide (CN) inhibition	100%	100%	[40]
Fe (atom/molecule)	156	4	[40]
Cu (atom/molecule)	3.58 ± 1.17	0	[40]
Zn (atom/molecule)	5.13 ± 0.75	0	[40]

**Table 3:** In vitro studies involving M101 application.

Application	Concentration & Study Type	Results	Conclusion	Reference
Human oral epithelial cells (EC), human oral fibroblasts, human osteoblasts, BALB/3T3 cells *P. gingivalis*	1 g/L. Cells were exposed to M101 for up to 24 h and cytocompatibility was determined. EC exposed to lipopolysachharide of *P. gingivalis (Pg-LPS)* or infected with *P. gingivalis* to mimic inflammatory state were treated with M101 and inflammatory markers were studied. *P. gingivalis* biofilms grown on glass surface were treated with M101.	No biologically significant reduction in cellular viability and metabolic activity in all the four cell types tested. ↘ pro-inflammatory markers’ release (TNF-α, NF-κB, RANKL, IL-1β, IL-8, RANTES, IP-10). ↗ anti-inflammatory and pro-resolution mediators (IL-2, IL-4, IL-10, IL-11, IL-15, PDGF-BB, TGF-β, Resolvin-E1 receptor) (*p* < 0.005). ↗ extracellular matrix and immune modulators (TIMP-2, M-CSF and ICAM-1) (*p* < 0.005). ↘ *P. gingivalis* growth.	M101 decreases *P. gingivalis*-induced inflammation.	[81]
Cold stored porcine proximal tubular cells line (kidney epithelial cells (LLC-PK1 cells)	M101 was supplemented (0–10 g/L) to University of Wisconsin (UW), histidine-tryptophane-ketoglutarate (HTK), Institut Georges Lopez IGL (IGL-1), Celsior, RingerLactate (RL) and Perfadex. LLC-PK1 cells or N2 gas was used to deoxygenate the solution. M101 SOD activity was evaluated by nitroblue tetrazolium assay (NBT).	Commercial preservative solutions: ↘ cell viability (LDH release); ↘ metabolic activity (MTT) and ATP content. M101 supplementation in commercial preservative solutions: ↗ cell integrity; ↗ cell functionality. Total inhibition of NBT formation with M101 (93.5 ± 1.1%).	Commercial preservative solutions alone were deleterious to cells. M101 protects cells in vitro against cold preservation lesions. M101 is an effective antioxidant.	[57]
Human primary aortic endothelium cells (HAECs)	In vitro model of cold hypoxia and reoxygenation simulating reperfusion on cold-preserved cells. HAECs were subjected to 24 h hypoxia at 4 °C in UW solution mimicking cold ischemia during organ preservation. M101 (0, 1, 2.5, 5, 10 g/L), was added to UW solution. UW was removed and cells were re-cultured at 37 °C to mimic organ reperfusion. Necrosis assay, lactate dehydrogenase (LDH) release.	*Cells after 24 h cold hypoxia:* Cells in UW alone: ↗ LDH released from necrotic cells (32%); ↘ intracellular ATP (47%); ↘ mitochondrial respiratory chain complex II activity. Cells M101 + UW: ↘ LDH; ↗ intracellular ATP; ↗ mitochondrial respiratory chain complex II. Cells in UW alone: ↗ necrotic bodies and myelinic figures; ↗ cell organelles altered and damaged (stress). Cells M101 + UW: ↘ necrotic debris; ↗ organelles integrity presence of autophagosomes rather than necrotic bodies. *Cells after 24 h simulated reperfusion:* UW alone: ↗ Cell injuries; ↗ LDH release (51%). ↘ intracellular ATP (24%); ↘ respiratory mitochondrial complex II activity (33%). Cells M101 + UW: ↘ LDH release (13%); ↗intracellular ATP (86%); ↗ metabolic activity (XTT) for the 5 g/L dose (62%) (*p* ≤ 0.05 vs. 0 g/L).	M101 imparts dose dependent protection against cold hypoxia. M101 conserves cell integrity during preservation. M101 provides dose-dependent protection against IRI.	[63]
In vitro Measurement of NO and CO Binding rates	Nitric oxide (NO) and carbon monoxide (CO) reaction kinetics measurements. Briefly, 3 mL of a deoxygenated PBS solution containing HbV, human HbA, Oxyglobin (Biopure) and M101 at (heme) = 3 µM was rapidly mixed with NO or CO containing PBS solution. Changes were recorded at 10, 30 and 60 min post-infusion.	M101 showed a lower binding rates of NO and CO than human Hb and polymerized bovine Hb.	M101 has different binding rates for gases as compared to human Hb.	[40]

**Note:** ↘ decrease; ↗ increase.

**Table 4:** Ex vivo, in vivo and clinical studies involving M101 use.

Application	Concentration &Study Type	Results	Conclusion	Reference
Isolated Langendorf-perfused rat hearts (*n* = 12/group)	Evaluation of M101 (1 g/L) as a protective additive to Celsior solution for static storage of donor hearts prior to transplantation. Heart function in Celsior solution, either alone (control) or with the addition of M101 was measured by intra-ventricular balloon before arrest with cold (7.5 °C). Cold storage (CS) lasted 8 h prior to reperfusion (60 min). Hearts (minced and homogenized) were also assessed by 2,3,5-triphenyltetrazoliumchloride (TTC) staining as a measure of viability and infarct size.	M101: ↗ recovery of left ventricular developed pressure. Recovered heart rate to pre-ischemic value (final recovery between 84 and 89% pre-ischemic value). ↗ coronary flow (7.5 ± 0.7 mL/min) compared to control (5.4 ± 0.4 mL/min) (*p* < 0.05). Viability and infarct size measurements were similar between groups.	The addition of M101 to Celsior preservation solution significantly improved post-ischemic recovery of heart function.	[62]
Rat pancreas preservation	Preservation solution with or without M101 at 2 g/L was injected into the pancreas via the pancreatic duct. Pancreas was removed and placed in the preservation solution at 4 °C, and cold ischemia kinetics were then determined. Samples were taken at 0, 2, 4, 6, 8, 12 or 18 h (*n* = 4–6). Metabolite extraction was performed on fresh tissue, while protein extraction and OCT slide analysis were performed on snap-frozen tissue at each time-point. Islet isolation was performed after cold ischemia (30 min, 4, 6, 8, 12 or 18 h). For the experiments with M101 perfusion, 2 mL of preservation solution with or without M101 at 2 g/L was injected into the pancreas via the pancreatic duct. Pancreases were preserved for 6 h at 4 °C in the presence of M101, before islet isolation process. 12 h of rat pancreas preservation in the presence of M101.	M101: ↗ maintenance of mitochondrial complex 1 pancreas activity throughout ischemia kinetics compared to controls; ↘ Lactate levels compared to the control; ↘ variability of active caspase-3 levels; ↘ transient phosphorylation of p38, which is observed in the control, after 4 h (*p* = 0.002) and 6 h (*p* = 0.008) of ischemia; ↘ oxidative stress ROS (25%) compared to control; ↘ p38 activity in islets; ↘ necrosis (HMGB1); ↘ cellular stress pathway (p38 MAPK) activity.	Safe, efficient, anti-oxidant. Freshly isolated islets had improved function when M101 injected in the pancreas.	[65]
Human pancreas	Evaluation of M101 effects during cold preservation. Human pancreases (*n* = 3) underwent 6 h of cold ischemia between removal from donors and arrival in our laboratory. M101 (2 g/L) was added to Belzer^®^ solution (UW solution, Bridge to Life) and then injected into the pancreas for 3 h. Protein extraction was performed on snap-frozen pancreatic tissue after 1 or 3 h of M101 exposure at 4 °C. Islets were isolated after 9 h of cold ischemia and counted after 12 h culture post-isolation in CMRL medium (Sigma-Aldrich) at 37 °C. Functionality of islets was assessed by a glucose-stimulated insulin secretion test. A Spearman’s correlation was performed to check the influence of age on IEQ or insulin secretion. Human pancreases were exposed to M101 for 3 h.	M101: ↘ HIF1-α expression in pancreas after 1 h of exposure, but not after 3 h; ↗ AKT phosphorylation in tissue after 3 h of exposure to M101 (*p* = 0.08); ↗ increased complex 1 activity; ↘ cleaved caspase-3 in tissue; ↗ islet yield and function of pancreases relative to the control. ↗ insulin secretion from islets (both in basal and in stimulated conditions); ↗ overall insulin content; ↗ complex 1 mitochondrial activity; ↗ activation of AKT activity (a cell survival marker); ↘ variability of caspase 3; ↘ oxidative stress (ROS); ↘ necrosis (HMGB1); ↘ cellular stress pathway (p38 MAPK) activity; ↗ islet yield and function.	Despite the absence of M101 during the first period of ischemia, positive effects were observed in human pancreas during preservation.	[65]
Pig pulmonary preservation and post-transplant lung function	36 h cold preservation with 1 g/L of M101. Normothermic ex vivo lung perfusion (EVLP) followed by lung transplant (4 h reperfusion).	M101-treated lungs improved physiologic parameters: ↗ oxygenation than in the controls significantly; ↘ edema formation significantly; ↘ circulating IL-6 within recipient plasma after transplantation.	M101 during an extended pulmonary preservation period led to significantly superior early post-transplant lung function.	[82]
Inflammatory calvarial abscess model in mice	M101 (1 g/L). *P. gingivalis* (5 × 10^8^ CFU) was injected subcutaneously on the calvaria to induce inflammatory abscess which was treated with M101 (1 g/L) subcutaneous injection. The healing response was followed for 5 days.	↘ clinical abscess size significantly; ↘ inflammatory score significantly; ↘ osteoclastic activity.	M101 decreased *P. gingivalis* elicited inflammation and tissue destruction.	[81]
Traumatic brain injury (TBI) rat	M101 (12.5 mL/kg I.V. over 2 h = 625 mg/kg cumulated doses); M101 (12 mL/kg I.V. over 1 h = 600 mg/kg).	M101 (12.5 mL/kg i.v. over 2 h): ↗ MAP (mild 9 mm Hg) of healthy rats without constriction of cerebral pial arterioles. M101 (12 mL/kg i.v. over 1 h): ↗ MAP (modest 27 mm Hg) (peak, 123 ± 5 mm Hg [mean ± standard error of the mean]) of CCI-TBI rats; ↗ PbtO_2_ brain tissue oxygen (peak, 25 ± 5 mm Hg), restored brain tissue oxygen to 86% of pre-injury level.	Proof of concept that M101 may be beneficial in TBI treatment	[39]
Mice: in vivo oxygenation potency of M101 toward HT29 human colorectal adenocarcinoma subcutaneous tumors	M101 I.V. injection at 600 mg/kg and 1200 mg/kg. Single I.V. injection of M101 in mice bearing human-derived subcutaneous tumors. Expression of hyperoxia marker anti-glucose transporter Glut-1 (immunohistochemistry).	Rapid diffusion of M101 in brain, liver, lungs and ovaries: ↘ size of carcinomatous areas; ↗ dissociation of tumor; ↘ intensity of Glut-1 staining ∼20% after 1 h when M101 was injected at 1200 mg/kg; ↘ tumor hypoxia of ∼23% (even 5 h after the same treatment); ↘ hypoxia in poorly vascularized tissues. Lower M101 doses (60 or 600 mg/kg), no or intermediate hypoxia reductions. No side effects.	Potential oxygen carrying therapeutic product. Ability of M101 to diffuse within poorly vascularized tissues and to behave as a potent O2 carrier towards vertebrate tissues, without inducing obvious side effects.	[38]
Static lung transplantation pigs	HEMO_2_ life added to preservative solution (Perfadex^®^). 24 h of hypothermic preservation followed by lung transplantation. Five hours of lung reperfusion.	HEMO_2_life group: ↘ graft vascular resistance (*p* < 0.05); ↗ graft oxygenation ratio (*p* < 0.05); ↘ serum HMG B1.	HEMO_2_life improved early graft function after prolonged cold ischemia.	[66]
Mice: testing immunogenicity of M101	Antibody response after 1 or 2 I.V. administrations of M101 in hyper-responsive strain (BP/2 mice) that easily produce antibodies. Plasma levels of IgE and IgG2a measured by ELISA. Comparison with a negative control groups (M101 buffer) and Ovalbumin as a positive control.	After single intravenous injection, M101 increased IgE levels very slightly, but this effect did not reach statistical significance when compared to the vehicle-treated group and on the contrary treatment with ovalbumin led to a 3.4-fold increase in IgE level. After two administrations of M101 7 days apart, a slight and non-significant increase of IgE and IgG2a levels was observed at D14 when compared to vehicle treated group. The second administration of M101 at D7 did not induce any mortality. On the contrary, in the ovalbumin-treated group, three mice out of nine died 15 min after the challenge at D7. The remaining mice showed a marked increase in IgE level after the second injection (D14).	M101 did not cause any immunogenic reaction.	[35]
Pig: kidney autotransplantation model	Four groups were studied for organ preservation and subsequent in vivo transplantation: 1. UW; 2. UW + M101 (5 g/L); 3. HTK: HTK; 4. HTK + M101 (5g/L). Controls were sham-operated animals. Animals were followed-up until the sacrifice at 3 months.	In M01 kidney grafts: ↗ resumption of urine production; ↗ kidney graft function recovery; ↗ preservation of tissue integrity; ↘ inflammation; ↘ invasion of both innate (ED1+) and adaptative (CD3+) immune cells; ↘ chronic fibrosis; ↘ tubular atrophy.	Beneficial use of M101 in 2 of the most used preservation solutions (better short-term function recovery and reduced development of fibrosis, the main cause of graft loss).	[57]
Pig: kidney autotransplantation model	Kidney was harvested, cold flushed and preserved for 24 h at 4 °C before transplantation. Dose-ranging study: 1, 2, and 5 g/L M101 supplementing UW solution. Pigs placed in metabolic cages for diuresis and serum creatinine measurements. Biopsies for conventional histology and fibrosis evaluated on sacrifice by Sirius red staining.	↘ intensity of IRI; ↘ interstitial fibrosis; ↗ graft outcome. Area under the curve of serum creatinine for the first 2 weeks after transplantation showed that the 1 g/L and 2 g/L groups were efficient and nearly identical.	HEMO2Life improved kidney graft function. Beneficial use of M101 at lower dosage.	[63]
Kidney machine preservation in a porcine transplantation model	Kidneys were submitted to 1 h-warm ischemia, followed by 23 h hypothermic preservation in Waves machine perfusion (MP) before auto-transplantation. Four groups were studied: 1. W (MP without 100%-O_2_); 2. W-O_2_ (MP with 100%-O_2_; also called hyperoxia); 3. W-M101 (MP without 100%-O_2_ + M101 2 g/L); 4. W-O_2_ + M101 (MP with 100%-O_2_ + M101 2 g/L).	First week post-transplantation, W-M101 group: ↘ renal resistance; ↘ blood creatinine; ↗ glomerular filtration rate; ↘ KIM-1; ↘ IL-18 blood levels; ↘ kidney fibrosis.	Supplementation with M101 associated with or without 100% O2 improved the Waves MP effect upon kidney recovery and late graft outcome.	[80]
Cold preservation in a preclinical porcine model of kidney donation after cardiac death	1 and 2 g/L M101. Comparison of addition of M101 in CS and MP (LifePort).	CS + M101: ↗ long term kidney function (dose dependent, on creatininemia after 1 and 3 month). Preservation in MP + M101: ↗ ATP content and VEGF expression; ↗ kidney function (normalized creatininemia time course and AUC); ↗ perfusion; ↗ preservation; ↘ kidney fibrosis.	In the CS arm, M101 improved long-term function, normalizing creatininemia. In the MP arm, M101 improved both short- and long-term functional outcomes, as well as tissue integrity. M101 is a viable strategy to improve current organ preservation method in marginal organ transplantation.	[79]
Pig liver orthotopic allotransplantation model	Addition of M101 to static cold storage (SCS) on the quality of pig liver graft preservation. Pig liver grafts were preserved using: 1. SCS; 2. Hypothermic oxygenated perfusion machine (HOPE); 3. SCS + M101 (1 g/L).	*HOPE or SCS+M101 compared with SCS alone:* After 9 h of ex vivo preservation, liver functions: ↗ mitochondrial function; ↗ATP synthesis; ↗ anti-oxidant capacities; ↗ hepatocyte architecture preservation; ↘ free radical production; ↘ inflammatory mediators. *HOPE ≥ SCS + M101 > SCS* After 6 h of ex vivo preservation and transplantation, liver functions: ↘ blood aspartate; ↘ blood alanine aminotransferases; ↘ blood LDH at day one post-transplant. At days 1 and 3: ↘ TNF-α levels. At day 7: ↘ liver cell necrosis; ↘ liver inflammation; *HOPE ≥ SCS + M101 > SCS*	When added to SCS, M101 effectively oxygenated liver grafts during preservation, preventing post-transplant injury without reaching the level of HOPE. This needs to be weighed against the cumbersomeness and cost of infusion machines, which require continuous presence of highly-skilled operators, and are associated with a risk of vascular trauma in organ grafts that might compromise the outcome of transplantation.	[64]
Hamster & rat cardio-vascular study	Hamster study: dorsal skinfold window chamber model to evaluate microcirculation Changes were recorded 60 min after M101 infusion. Rat study: to evaluate cardiovascular effects. M101 (600 mg/kg) or saline solution 0.9% (control). Changes were recorded 45 min after M101 infusion.	In hamster: absence of microvascular vasoconstriction and no significant effect on mean arterial blood pressure. In rat: minor effects on mean arterial pressure (differences not statistically significant compared to control), heart rate and myocardial contractility.	M101 appears to have no vasoactivity at the microvascular level.	[40]
Human safety and proof-of-principle study	First-in-human use of M101 (1 g/L) for organ preservation. Open-label study investigating the safety of M101 used ex vivo as an additive to the preservation solution in kidney transplantation (Clinical Trial Registry No. NCT 02652520). Grafts were preserved either in cold storage (standard donor) or on machine perfusion (extended criteria donor).	60 graft kidneys from 60 deceased donors were preserved with M101. M101 is safe for the graft and for the recipient. No allergic or hypersensitivity reactions or infections related to the product were reported. Less DGF (at least one dialysis) in the M101 group was reported, and the difference between the two groups (23% vs. 33%) was clinically relevant but not statistically significant. When a more stringent definition (more than one dialysis) is used, the difference (7% vs. 26%) was statistically significant (*p* = 0.038).	This study demonstrated that the addition of the oxygen carrier M101 to preservation solution is safe. Although the study was not designed to show the superiority of M101, the analysis of the secondary efficacy end points is highly promising, with better renal function in recipients of the kidneys preserved with M101.	[51]
